# Identifying possible asthma–COPD overlap syndrome in patients with a new diagnosis of COPD in primary care

**DOI:** 10.1038/npjpcrm.2016.84

**Published:** 2017-01-05

**Authors:** Camilla Boslev Baarnes, Peter Kjeldgaard, Mia Nielsen, Marc Miravitlles, Charlotte Suppli Ulrik

**Affiliations:** 1 Department of Pulmonary Medicine, Hvidovre Hospital, Hvidovre, Denmark; 2 Department of Pneumology, Hospital Universitari Vall d’Hebron, Barcelona, Spain; 3 Institute of Clinical Medicine, University of Copenhagen, Copenhagen, Denmark

## Abstract

The asthma–chronic obstructive pulmonary disease (COPD) overlap syndrome (ACOS) remains poorly characterised. Our aim was to describe an algorithm for identifying possible ACOS in adults with newly diagnosed COPD in primary care. General practitioners (*n*=241) consecutively recruited subjects ⩾35 years, with tobacco exposure, at least one respiratory symptom and no previous diagnosis of obstructive lung disease. Possible ACOS was defined as chronic airflow obstruction, i.e., post-bronchodilator (BD) forced expiratory volume 1/forced vital capacity (FEV_1_/FVC) ratio<0.70, combined with wheeze (ACOS wheeze) and/or significant BD reversibility (ACOS BD reversibility). Of 3,875 (50% females, mean age 57 years) subjects screened, 700 (18.1%) were diagnosed with COPD, i.e., symptom(s), tobacco exposure and chronic airflow obstruction. Indications for ACOS were found in 264 (38%) of the COPD patients. The prevalence of ACOS wheeze and ACOS BD reversibility was 27% (*n*=190) and 16% (*n*=113), respectively (*P*<0.001), and only 6% (*n*=39) of the COPD patients fulfilled both criteria for ACOS. Patients with any ACOS were younger (*P*=0.04), had more dyspnoea (*P*<0.001), lower FEV_1_%pred (67% vs. 74%; *P*<0.001) and lower FEV_1_/FVC ratio (*P*=0.001) compared with COPD-only patients. Comparing subjects fulfilling both criteria for ACOS with those fulfilling criteria for ACOS wheeze only (*n*=151) and those fulfilling criteria for ACOS BD reversibility only (*n*=74) revealed no significant differences. Irrespective of the applied ACOS definition, no significant difference in life-time tobacco exposure was found between ACOS- and COPD-only patients. In subjects with a new diagnosis of COPD, the prevalence of ACOS is high. When screening for COPD in general practice among patients with no previous diagnosis of obstructive lung disease, patients with possible ACOS may be identified by self-reported wheeze and/or BD reversibility.

## Introduction

A significant proportion of patients presenting with symptoms of obstructive lung disease has features of both asthma and chronic obstructive pulmonary disease (COPD),^1,2^ often referred to as the asthma–COPD overlap syndrome (ACOS).^3–5^ In recent years, the ACOS has gained much attention and been extensively reviewed.^3,5–9^ However, so far there is no generally agreed term or defining features for this category of patients with chronic airway obstruction,^10^ although diagnostic criteria have been proposed based on consensus for overlap in patients already diagnosed with COPD.^4,11,12^

The proportion of patients with ACOS among individuals with existing COPD is unclear, as it depends very much on the applied defining criteria, but has been reported to be between 15 and 60%.^13–15^ Furthermore, concurrent doctor-diagnosed COPD and asthma have been reported in up to 20% of patients with obstructive lung disease.^16,17^ Given these uncertainties, it appears important to establish useful and reliable criteria for identifying patients with possible ACOS, i.e., an asthma component of their disease, not least when these patients are diagnosed in primary care.

Pharmacological therapy of patients with asthma–COPD overlap can at present not be evidence-based, as this group of patients has, although together with a substantial proportion of all patients with obstructive lung disease, consistently been excluded from participating in clinical trials.^1^ So, as clinical trials have until now only enrolled patients with the extreme phenotypes of both asthma and COPD,^18^ there is a clear need for clinical trials to establish evidence-based therapy for this group of patients.^6^ In the meantime, a number of national guidelines^11,12,19^ and the GINA-GOLD ACOS document^4^ have attempted to establish consensus-based treatment options for patients with ACOS. However, as patients with asthma–COPD overlap seem to be at risk for a poor outcome, including a high risk of exacerbations, it is important to identify this subgroup of patients with COPD to ensure adequate treatment of the asthma component of their disease, including anti-inflammatory therapy, also for patients diagnosed in primary care.

The aim of the present study was to describe an algorithm for identifying possible ACOS in adults with newly diagnosed COPD by applying different diagnostic criteria in a large cohort of individuals at high risk of COPD, but no previous diagnosis of obstructive lung disease, in a primary care setting to facilitate identification of patients with an asthma component of their disease, as this subgroup of patients with COPD is likely to be at risk for a poor outcome.

## Results

### Description of cohort

#### Baseline characteristics of enrolled subjects

A total of 241 general practitioners (GPs; ~7% of Danish GPs) participated in the study. Of the 4,049 screened subjects, 3,875 (95.7%; 50% males; mean age 57 years (range 35–92 years)) fulfilled the inclusion criteria, and were included in the present analysis. Of the enrolled subjects, 2,390 (61.7%; no difference between males and females) were current smokers (mean estimated life-time tobacco exposure 34.5 pack-years) and 1,485 ex-smokers (38.3%, mean 28.5 pack-years; Table 1).

#### Prevalence of respiratory symptoms and airflow obstruction

**Figure 1:**
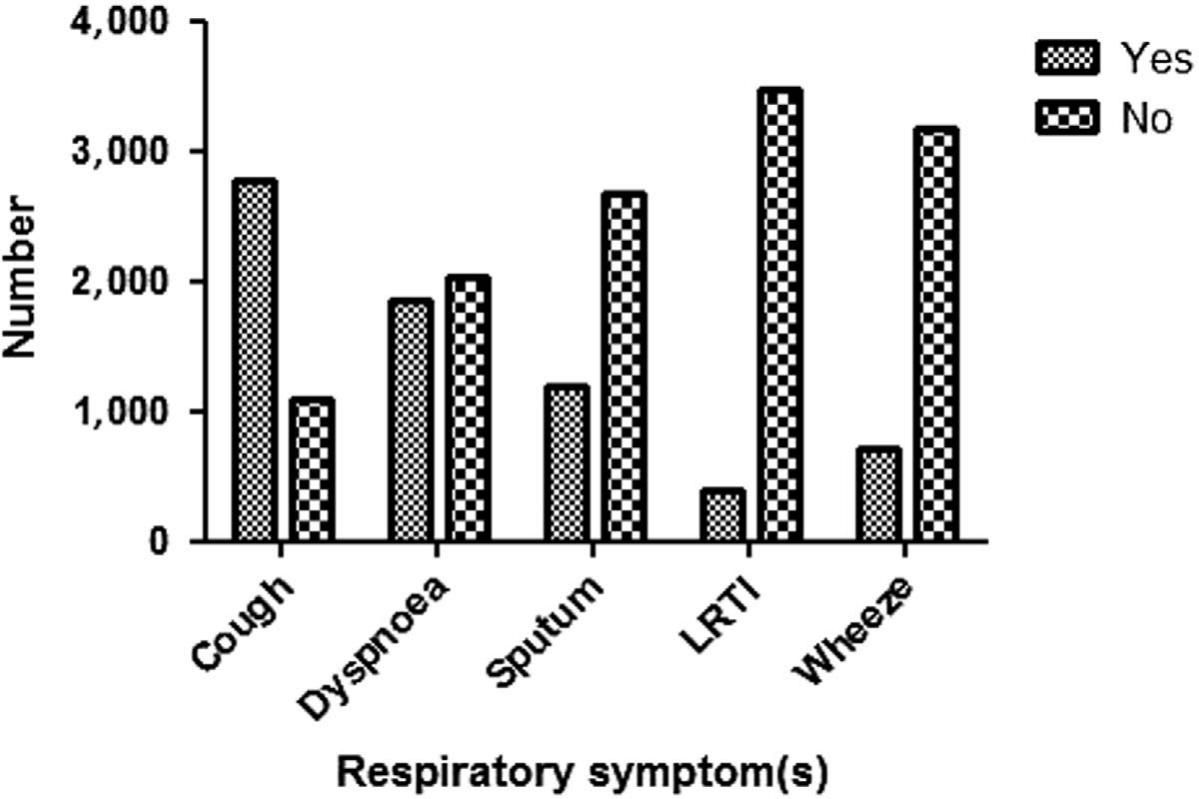
Prevalence of respiratory symptoms among the enrolled subjects (*n*=3.875). LRTI, recurrent lower respiratory tract infections.

Cough was the most prevalent symptom among the enrolled subjects, followed by dyspnoea and sputum production (Figure 1).

Of the 3,875 subjects enrolled in the study, 700 (18.1%) fulfilled the criteria for chronic airflow obstruction, i.e., a post-bronchodilator (BD) forced expiratory volume 1/forced vital capacity (FEV_1_/FVC) ratio<0.70 (Table 1). Furthermore, 1.190 (30.7%) of the enrolled subjects had an FEV_1_<80%pred.

Subjects with self-reported wheeze (*n*=710; 18.3%) had, compared with participants not reporting wheeze, a significant higher prevalence of both chronic airflow obstruction (27.1% vs. 16.4%; *P*<0.001) and FEV_1_<80%pred (44.9% vs. 27.5%; *P*<0.001).

### Non-COPD participants

A total of 3,175 (77%) of the subjects had tobacco exposure and at least one respiratory symptom, but no chronic airflow obstruction, i.e., FEV_1_/FVC ratio>0.70 either at the screening spirometry (*n*=2,963) or following the administration of BD (*n*=212). Of these 3,175 subjects, 718 (23%) had an FEV_1_<80%pred.

### Participants with COPD

Of the 700 patients fulfilling the diagnostic criteria for COPD, 85% had, based on spirometric criteria, mild-to-moderate COPD (Table 1). Patients diagnosed with COPD were older, had more dyspnoea (Medical Research Council (MRC) score) and higher life-time tobacco exposure (39.7 and 30.5 pack-years, respectively; *P*<0.0001) compared with non-COPD participants (Table 1).

#### Any ACOS defined as chronic airflow obstruction and wheeze and/or a positive BD test compared with COPD only

**Figure 2:**
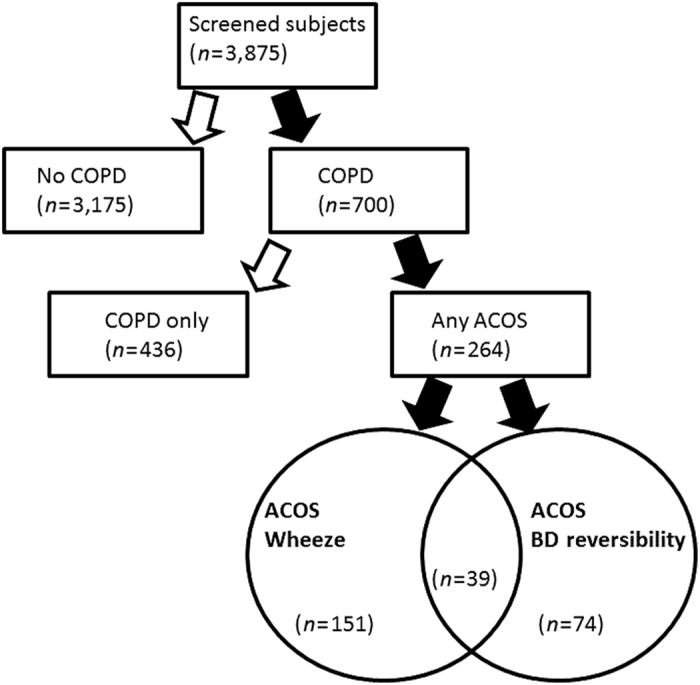
Flow chart of the 3,875 individuals at high risk of COPD, but no previous diagnosis of obstructive lung disease, included in the present analysis divided according to the presence or absence of COPD, possible asthma–COPD overlap syndrome (ACOS) or COPD only, respectively.

The prevalence of any ACOS among all screened subjects (*n*=3,875) was 6.8%. Any ACOS was found in 264 (37.8%) of the patients with a new diagnosis of COPD (Figure 2).

Compared with patients with COPD only, patients with any ACOS were characterised by being younger, having more dyspnoea (MRC score), lower FEV_1_ and lower FEV_1_/FVC ratio (Table 2), whereas no difference was found in life-time tobacco exposure. In line with this, patients fulfilling both criteria for ACOS were also younger (*P*<0.05), had more dyspnoea (*P*=0.001), lower FEV_1_ (*P*=0.01) and lower FEV_1_/FVC ratio (*P*<0.001) compared with COPD-only patients, but no difference in number of pack-years (*P*=0.75).

#### ACOS defined as chronic airflow obstruction and wheeze (ACOS-W)

The prevalence of ACOS defined as chronic airflow obstruction and wheeze, i.e., ACOS-W, in the entire cohort was 5% (*n*=190) corresponding to a prevalence of ACOS-W of 27.2% within the group of subjects diagnosed with COPD (Table 3). Patients with ACOS-W had, as assessed by the MRC score, more dyspnoea (*P*<0.0001) compared with subjects with COPD not reporting wheeze (*n*=510).

Comparing patients with ACOS-W and COPD only showed that the former subjects were younger (61.5 vs. 63.5 years; *P*=0.02) and had higher body mass index (26.8 vs. 25.4; *P*<0.01), lower post-BD FEV_1_/FVC ratio (0.60 vs. 0.62; *P*=0.02) and lower post-BD FEV_1_%pred (65.7 (18.7) vs. 73.1 (18.9); *P*<0.0001), whereas no significant difference was found in life-time tobacco exposure (pack-years) and absolute or percentage increase in FEV_1_ after administration of BD.

#### ACOS defined as chronic airflow obstruction and positive BD reversibility test

One-hundred and thirteen subjects (3% of the entire cohort) had chronic airflow obstruction and a positive BD reversibility (BDR) test, i.e., ACOS-BDR. The prevalence of ACOS-BDR was 16.2% among the patients diagnosed with COPD (Table 3). No significant difference was found in MRC score between patients with COPD only and ACOS-BDR (*P*=0.6).

Patients with ACOS-BDR had significantly lower FEV_1_/FVC ratio (0.59 vs. 0.61; *P*=0.03) and, by definition, higher BDR (*P*<0.001) compared with patients with COPD only, whereas no significant difference was found between the two groups in age, body mass index, pack-years of smoking or FEV_1_%pred.

#### ACOS defined as chronic airflow obstruction and wheeze and a positive BD test compared with ACOS-W and ACOS-BDR only

A total of 39 patients (5.6%) diagnosed with COPD fulfilled both criteria for ACOS; 151 and 74 patients, respectively, only fulfilled the ACOS wheeze and ACOS-BDR criteria (Figure 2). Subjects fulfilling both criteria for ACOS had more dyspnoea compared with both ACOS-W- and ACOS-BDR-only patients (*P*<0.01 for both comparisons), but no differences were found between the groups, i.e., ACOS-W+ACOS-BDR versus ACOS-W only and ACOS-BDR only, with regard to age, number of pack-years, FEV_1_%pred or FEV_1_/FVC ratio.

**Figure 3:**
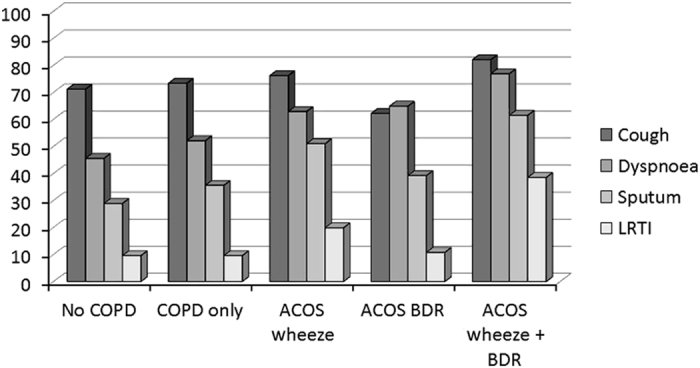
Prevalence of respiratory symptoms among participants with no COPD (*n*=3.175), COPD only (*n*=436) and possible ACOS, identified by either self-reported wheeze (ACIS wheeze; *n*=151), bronchodilator reversibility (ACOS-BDR; *n*=74) of both (ACOS wheeze+BDR; *n*=39).

The prevalence of respiratory symptoms, other than wheeze, in the groups of subjects classified as having possible ACOS (as defined above) compared with participants with no COPD and COPD only is given in Figure 3. In general, patients with possible ACOS had more symptoms than participants with no COPD and COPD only, whereas no gender differences were found.

## Discussion

### Main findings

In the present study, we investigated algorithms for identifying possible ACOS in a large cohort of subjects at high risk of COPD (prevalence of COPD 18.1%), but with no previous diagnosis of obstructive lung disease, including asthma, aiming at facilitating the identification of COPD patients with an asthma component of their disease.

Among the 700 (18.1% of the cohort) patients diagnosed with COPD, the prevalence of ACOS varied between 5.6 and 27.2% depending on the applied criteria. The combination of chronic airflow obstruction and wheeze as criteria revealed the highest prevalence of possible ACOS (27%), and compared with COPD only, these patients had more dyspnoea and lower FEV_1_%pred, whereas no difference was found in BDR. In contrast to this, these differences were not found between patients with possible ACOS- and COPD-only patients when ACOS was defined on the basis of BDR (ACOS-BDR). Patients with COPD fulfilling the applied definition of ACOS, irrespective of the criteria, had significantly lower FEV_1_/FVC ratio compared with the COPD-only patients, whereas no differences were found in life-time tobacco exposure when comparing any ACOS and COPD only.

### Interpretation of findings in relation to previously published work

No universal consensus exists on diagnosis of asthma–COPD overlap,^9,20^ and we, therefore, based our classification on elements from the Global Initiative for Asthma and Global Initiative for Chronic Obstructive Lung Disease document^4^ together with criteria applied in previously published studies.^21–23^ Furthermore, as our primary aim was to develop an algorithm for identifying individuals with possible ACOS among patients with a new diagnosis of COPD in general practice, the applied criteria had to be obtainable in that setting. Self-reported wheeze has been shown to be far more prevalent among patients classified as having asthma–COPD overlap compared with COPD only^24^ and the BDR criteria has been recommended and applied in previous studies.^4,21,23,25^

The average tobacco exposure was found to be 32 pack-years, which is lower than that often reported from clinical trials of patients with COPD,^26,27^ most likely due to the selection of subjects with no previous diagnosis of obstructive lung disease; and in keeping with previous studies, tobacco exposure was higher, and level of lung function tended to be lower, in males compared with that in females. Another important difference between the present cohort and patients enrolled in COPD trials is the high proportion of current smokers (62%) in our study, which points to an important opportunity for guidance on smoking cessation, also because the most common respiratory symptom was cough, by many smokers regarded as a harmless smoking-related symptom.

Of the enrolled subjects without chronic airflow obstruction (*n*=3,175), 23% had lung function impairment, i.e., FEV_1_<80%pred that may suggest underlying respiratory disease other than COPD, but, unfortunately, the present diagnostic algorithm did not allow us to evaluate these individuals further.

The highest prevalence of possible ACOS was observed by applying the definition of chronic airflow obstruction and wheeze. Wheeze is caused by air passing through too narrow airways, and may be caused by different disease processes, including asthma, COPD and heart failure. Thus, this definition may, therefore, seem too broad for identifying individuals with an overlap between asthma and COPD. In keeping with this, patients classified into the ACOS group by this definition had more dyspnoea compared with the COPD-only group. Using the same criteria, although in a cross-sectional population study, Chung *et al.*^28^ found a prevalence of ACOS of 2.3% in the total population, corresponding to a prevalence of 30% in the COPD group, similar to the findings in the present study. Furthermore, Huang *et al.*^29^ have recently reported that the presence of episodic wheezing in patients with COPD has a negative impact on morbidity.

Previously, studies have reported a prevalence of ACOS from 15 to 60% among patients with COPD.^13–15^ However, the criteria for ACOS differs between studies,^30^ as some are based on symptoms and spirometric parameters,^28,31^ others on the presence of a physician’s diagnosis of both asthma and COPD,^13,32–34^ and some on a mixture of these criteria.^14,35^

The present opportunistic screening study is, to our knowledge, the first to apply different definitions of ACOS in the same cohort of individuals with newly diagnosed COPD, and by that demonstrating a wide variation in proportion of patients who will be classified as having ACOS among patients with COPD depending on the applied diagnostic criteria. However, as the patients enrolled in the present study had no previous diagnosis of obstructive lung disease, direct comparisons with previous studies are difficult.^5,35^ However, in the study by Menezes *et al.*^35^ ACOS was defined as a FEV_1_/FVC<0.7 plus wheezing in the past 12 months plus BDR, revealing a prevalence of ACOS of 13% (89 patients) among their 683 COPD patients, which is higher than the 5.6% observed in our study. However, comparable to our study, they found a higher degree of reversibility, but no difference in estimated life-time tobacco exposure. Furthermore, in contrast to our findings, their patients with ACOS were slightly younger, had higher body mass index and lower FEV_1_ compared with COPD-only patients, and experienced more dyspnoea.

### Strengths and limitations of the study

As there is no gold standard for the diagnosis of ACOS,^9,36^ we cannot be absolutely sure that newly diagnosed COPD patients with BDR and/or self-reported wheezing always have ACOS, nor that patients with these characteristics will be considered to have ACOS by all examining physicians. The latter not least because it has been recognised for years, and reported from large-scale clinical trials, that patients with COPD may have reversibility without being considered to have an asthma component of their disease.^37^ However, in spite of that, the present study may provide important knowledge with regard to identification of patients with possible ACOS among new patients diagnosed with COPD.

The majority of patients with COPD are diagnosed and managed in primary care, and as wheezing and BDR are easy to detect characteristics also in primary care,^38^ our findings may offer substantial guidance for general practitioners to screen for possible ACOS among their patients with COPD, although further diagnostic work-up is likely to be needed. By this, our findings add to a recent study showing that a previous diagnosis of asthma may also be a reliable criterion for a probable diagnosis of ACOS.^39^ However, one of the main differences between the two studies is that patients with a previous diagnosis of asthma were excluded from the present study.

### Implications for future research, policy and practice

The prevalence of possible ACOS was high in our subjects with a new diagnosis of COPD (5.6–27.2%), irrespective of the applied definition. Our findings, therefore, suggests a substantial occurrence of ACOS among subjects with COPD identified by opportunistic screening in general practice, which points to the GP as a key person in identifying subjects with ACOS; not least because these subjects are likely to have a high risk of poor outcome, especially if they are not prescribed adequate treatment for the asthma component of their disease, including anti-inflammatory therapy.

The present observations are likely to have important implications not only for management, including pharmacological therapy, but also for outcome for patients with newly diagnosed COPD. However, beforehand it seems of utmost importance to reach consensus with regard to diagnostic criteria, as this is a prerequisite for clinical trials of therapeutic options and, later, the development of evidence-based guidelines for the management for this group of patients.

### Conclusions

The present study showed that a large proportion of subjects with a new diagnosis of COPD, in the majority of cases mild-to-moderate disease, can, irrespective of the applied criteria, be classified as having probable ACOS.

## Materials and methods

### Study design

GPs all over Denmark were invited to take part, and the aim was to engage at least 200 GPs (>5% of Danish GPs) to obtain a representative sample.^40^ Each of the participating GPs was expected to assess at least 20 consecutive subjects who attended their practice for respiratory or non-respiratory symptoms and fulfilled the criteria for participation in the study (6-month study period). Subjects included had all study-related procedures, including spirometry, performed in their own GPs practice (by trained staff).

### Material

Subjects were eligible for the study if they had no previous diagnosis of obstructive lung disease (COPD and/or asthma, including a history of early-onset asthma) provided they also fulfilled the following inclusion criteria: (1) age ⩾35 years, (2) smoker/ex-smoker, (3) ⩾1 respiratory symptom (dyspnoea, cough, wheeze, sputum and/or recurrent chest infections); and not the following exclusion criteria: (1) unable to perform spirometry.

### Methods

All participants filled in a questionnaire regarding age, gender, height, body weight, smoking status (including daily tobacco consumption and years of smoking), current airway symptoms (including cough, dyspnoea, wheezing, sputum and recurrent lower airway infections) and severity of dyspnoea (MRC scale).^41^ Spirometry was performed in accordance with the guidelines from the Danish Respiratory Society,^42^ and included at least three forced expiratory manoeuvres (and with the two highest measurements of FEV_1_ and FVC, respectively, differing <5%).

### Diagnostic algorithm

Airway obstruction was defined as FEV_1_/FVC ratio<0.70, in accordance with the GOLD strategy document.^43^ All participants with airway obstruction at screening spirometry (i.e., pre-BD spirometry) had a BDR test performed with 0.4 mg inhaled salbutamol (or equivalent) followed by a spirometry 15 min after; and only participants with a post-BD FEV_1_/FVC<0.70 were defined as having airway obstruction. A positive BD test was defined as an increase in FEV_1_>12% and 200 ml.^44^

### Definitions

COPD was diagnosed on the basis of current or previous tobacco exposure, respiratory symptom(s) and post-BD FEV_1_/FVC ratio<0.70, in accordance with the GOLD.^44^ Within the group of individuals diagnosed with COPD, ACOS was defined on the basis of criteria used in previous publications as follows: (1) self-reported wheeze^28,35^ (ACOS-W) and/or (2) a positive BDR test^35^ (ACOS-BDR).

### Data handling and analysis

Questionnaires and spirometric data were recorded in a consolidated web-based database. Derived values were automatically calculated by the computer, including number of pack-years, body mass index, FEV_1_% predicted and FEV_1_/FVC. Statistical analyses were performed with the software SPSS v. 21.0 (IBM corporation, Armonk, NY, USA).

The analyses were limited to subjects with complete data. Data were tested for normality, and non-parametric tests for independent samples were used to analyse continuous data. Categorical data were analysed by the Mann–Whitney *U*-test. In all the statistical analyses, a two-tailed *P*-value of ⩽0.05 was considered significant. Mean values are reported with s.d. Groups of interest were compared based on the presence of airway obstruction at screening spirometry, symptoms, MRC, COPD, ACOS and BDR.

### Ethics statement

The present study was endorsed by the Danish College of General Practitioners. According to the European Federation of Pharmaceuticals Industries and Associations code and the Danish Association of the Pharmaceutical Industry (LiF), the present study was a non-drug, non-interventional study, and approval from the scientific ethical committee and the Danish Medicines Agency was not mandatory, but they were given all relevant study information. The study was approved by the Danish Data Protection Agency.

## Figures and Tables

**Table 1:** Baseline characteristics of the enrolled subjects (*n*=3,875), including divided according to COPD status

	*All (*n*=3,875)*	*COPD (*n*=700)*	*No COPD (*n*=3,175)*
Age (years)	57.4 (11.8)	63.0 (10.5)	56.2 (11.7)^a^
BMI	27.0 (5.1)	25.8 (5.1)	27.2 (5.0)^a^
Pack-years	32.2 (22.3)	39.7 (23.2)	30.5 (21.8)^a^
FEV_1_ (l)	2.64 (0.88)	1.90 (0.69)	2.80 (0.83)
FEV_1_ (%pred.)	88.6 (19.6)	71.1 (19.1)	92.5 (17.5)
FEV_1_/FVC	0.75 (0.09)	0.61 (0.07)	0.79 (0.06)
MRC score	1.7 (0.7)	2.0 (0.8)	1.7 (0.7)^a^

Abbreviations: BMI, body mass index; COPD, chronic obstructive pulmonary disease; FEV_1_/FVC, forced expiratory volume 1/forced vital capacity; MCR, Medical Research Council.

a *P*<0.001 COPD versus no COPD.

**Table 2:** Characteristics of the 700 patients with a new diagnosis of COPD divided into participants with possible ACOS and COPD only

	*Any ACOS (*n*=264)*	*COPD only (*n*=436)*	P*-value*
Age (years)	61.9 (10.9)	63.6 (10.2)	*P*=0.04
BMI	26.2 (5.7)	25.5 (4.6)	NS
Pack-years	39.9 (22.8)	39.5 (23.4)	NS
FEV_1_ (l)	1.75 (0.67)	1.99 (0.68)	*P*<0.001
FEV_1_ (%pred.)	66.6 (18.7)	73.8 (18.8)	*P*<0.001
FEV_1_/FVC	0.60 (0.08)	0.62 (0.07)	*P*<0.001
MRC score	2.2 (0.8)	1.8 (0.8)	*P*<0.001
BD reversibility (l)	0.18 l (0.22)	0.03 (0.14)	*P*<0.001

Abbreviations: ACOS, asthma–COPD overlap syndrome; BD, bronchodilator; BMI, body mass index; COPD, chronic obstructive pulmonary disease; FEV_1_/FVC, forced expiratory volume 1/forced vital capacity; MCR, Medical Research Council.

**Table 3:** Characteristics of the 264 patients with possible ACOS, divided according to the presence of self-reported wheeze (ACOS wheeze) and/or a positive bronchodilator reversibility test (ACOS BD reversibility)

	*ACOS wheeze and ACOS BD reversibility (*n*=39)*	*ACOS wheeze (*n*=151)*	*ACOS BD reversibility (*n*=74)*
Age (years)	60.4 (12.0)	61.5 (10.9)	62.2 (11.2)
BMI	25.3 (5.6)	26.8 (5.9)	25.0 (5.1)
Pack-years	38.4 (18.3)	40.4 (22.6)	38.5 (21.9)
FEV_1_ (l)	1.71 (0.70)	1.79 (0.67)	1.64 (0.68)
FEV_1_ (%pred.)	66.4 (16.4)	65.7 (18.7)	68.0 (17.9)
FEV_1_/FVC	0.57 (0.08)	0.60 (0.08)	0.59 (0.08)
MRC score	2.5 (1.1)^a^	2.3 (0.9)^a^	2.1 (0.9)^a^

Abbreviations: ACOS, asthma–COPD overlap syndrome; BD, bronchodilator; BMI, body mass index; COPD, chronic obstructive pulmonary disease; FEV_1_/FVC, forced expiratory volume 1/forced vital capacity; MCR, Medical Research Council.

a *P*<0.01 for ACOS wheeze and BD reversibility compared with ACOS wheeze and ACOS BD reversibility.
